# Public health research priorities for WHO on COVID-19 in the South-East Asia Region: results of a prioritization survey

**DOI:** 10.1186/s12961-022-00862-x

**Published:** 2022-09-05

**Authors:** Tasnim Azim, Anjana Bhushan, Victor J. Del Rio Vilas, Rahul Srivastava, Pushpa Ranjan Wijesinghe, Roderico Ofrin, Sharat Chauhan, Anand Krishnan

**Affiliations:** 1 World Health Organization, South-East Asia Regional Office, New Delhi, India; 2 World Health Organization, New Delhi, India; 3 Centre for Community Medicine, All India Institute of Medical Sciences, New Delhi, India

**Keywords:** COVID-19, Research, Prioritization, Health system, WHO SEAR

## Abstract

**Background:**

Effectively addressing the coronavirus disease 2019 (COVID-19) pandemic caused by the new pathogen requires continuous generation of evidence to inform decision-making. Despite an unprecedented amount of research occurring globally, the need to identify gaps in knowledge and prioritize a research agenda that is linked to public health action is indisputable. The WHO South-East Asia Region (SEAR) is likely to have region-specific research needs.

**Methods:**

We aimed to identify a priority research agenda for guiding the regional and national response to the COVID-19 pandemic in SEAR countries. An online, anonymous research prioritization exercise using recent WHO guidance was conducted among the technical staff of WHO’s country and regional offices engaged with the national COVID-19 response during October 2020. They were each asked to contribute up to five priority research ideas across seven thematic areas. These research ideas were reviewed, consolidated and scored by a core group on six parameters: regional specificity, relevance to the COVID-19 response, feasibility within regional research capacity, time to availability for decision-making, likely impact on practice, and promoting equity and gender responsiveness. The total scores for individual suggestions were organized in descending order, and ideas in the upper tertile were considered to be of high priority.

**Results:**

A total of 203 priority research ideas were received from 48 respondents, who were primarily research and emergency response focal points in country and regional offices. These were consolidated into 78 research ideas and scored. The final priority research agenda of 27 items covered all thematic areas—health system (n=10), public health interventions (n=6), disease epidemiology (n=5), socioeconomic and equity (n=3), basic sciences (n=1), clinical sciences (n=1) and pandemic preparedness (n=1).

**Conclusions:**

This exercise, a part of WHO’s mandate to “shape the research agenda”, can help build a research roadmap ensuring efficient use of limited resources. This prioritized research agenda can act as a catalyst for Member States to accelerate research that could impact the COVID-19 response in SEAR.

## Background

The coronavirus disease 2019 (COVID-19) pandemic caused by a novel pathogen presents an unprecedented challenge to existing scientific knowledge and national responses. The pandemic has elicited a fundamentally multifaceted and evolving policy response from national governments addressing health, legal, social and economic issues in the immediate, short and long term [1, 2]. Updated scientific evidence can assist decision-making during public health emergencies to improve outcomes and reduce morbidity and mortality and the cost of relief efforts [3].

Knowledge generation is essential for ensuring evidence-informed programmes and policies. An enormous amount of research has been carried out and new knowledge gained on this new disease [4]. An initial assessment of studies on COVID showed that many of them were of low quality [5]. Poor quality of evidence usually, though not always, translates into lower strength of recommendations, which may have serious public health implications [6]. This creates problems for decision-makers, as they are unable to weigh the evidence or identify research that is relevant to them [7]. In this context, research should be undertaken such that the findings have local relevance and can be conducted within the available research capacity. While multiple types of evidence may be needed during a public health emergency, the urgency and the limited resource availability necessitate prioritization to enable targeted responses that will deliver the maximum impact.

Understanding this need, several COVID-19-related research prioritization exercises have been carried out [8–13]. Most of these were done in the early phase of the pandemic and were mainly by and for academicians and focused on clinical and epidemiological aspects in order to understand the disease process and its transmission. Later, as the pandemic spread and its impact became wider, researcher focus shifted to specific programme areas, such as maternal and child health, environment, mental health and ageing, to name but a few [14–17]. Also, the list of unanswered questions indicated a shift towards health system and public health interventions [18].

While global stakeholders address issues of global importance (which may also have regional relevance), regional resources are best spent on addressing issues which have higher regional relevance, though they may also be useful globally. Regional specificity may be needed where the results of global research are not applicable to the region or the methodology requires regional adaptation. For example, given the differences in the household structure and social dynamics, the results of household transmission studies from other parts of the world may not be applicable to countries of South-East Asia. It is also possible that some regional issues may not be addressed otherwise; for example, the role of traditional medicine in COVID-19 prevention and treatment. Finally, regional research priorities must consider the regional resources and capacity to undertake prioritized research.

One of WHO’s six core functions is to “shape the research agenda and stimulate the generation, translation and dissemination of valuable knowledge”. This core function is reflected in *The WHO strategy on research for health* and the WHO Regional Office for South-East Asia (SEARO) *Regional strategy on research for health 2018–2022* [19, 20]. Both documents identify research prioritization as an important area of the WHO mandate. In keeping with this mandate, SEARO carried out an exercise to identify the current research priorities that would be useful to guide regional and national responses to the current COVID-19 pandemic in the South-East Asia Region (SEAR).

### Methods

This exercise was designed, coordinated and managed by a core team in WHO/SEARO, guided by SEARO senior management. The core team was selected to represent different departments within WHO to gain a wider perspective. The core team had members from research, gender and equity, health emergencies, programme management and infectious hazard management backgrounds.

The exercise followed the recently released WHO guidance document for undertaking a research priority-setting exercise [21]. In addition, we followed the Child Health and Nutrition Research Initiative (CHNRI)-recommended principle of “wisdom of crowds” by independent scoring of research ideas by experts and the formation of a core coordination team to determine the scope and context of exercise and the criteria for prioritization [22]. The exercise was designed to put country priorities first, work for equity in development and link research to action, which are principles defining the essential national health research (ENHR) paradigm [23].

The core team brainstormed the scope and approach of the exercise. The exercise comprised four steps—preparatory work, generation of a preliminary list of research ideas, review and consolidation of these ideas, and the prioritization step.

### Preparatory work

As the objective of the exercise was the identification of regional priorities for the WHO SEARO and WHO country offices (WCOs) in Bangladesh, Bhutan, Democratic People’s Republic of Korea, India, Indonesia, Maldives, Myanmar, Nepal, Sri Lanka, Thailand and Timor-Leste, the intended participants in the prioritization exercise were all technical staff in these offices actively engaged with the national and regional COVID-19 response. It was understood that their input would be informed by and reflect the respective national COVID-19 research gaps and response priorities. Based on the initial review of earlier prioritization exercises referred to above, seven broad thematic areas were identified (Table 1).

**Table 1:** Thematic areas of research on COVID-19 for prioritization

Thematic area	Research that	Subthemes
Epidemiology	Improves our ability to monitor and predict the epidemic and provides evidence to inform public health interventions	•Burden, risk factors •Dynamics of disease transmission
Clinical	Helps describe the full clinical spectrum of COVID-19 including its impact on other organs, and improves diagnosis, chemoprophylaxis and clinical management of patients	•Clinical characteristics and management •Therapeutics and diagnostics •Impact on mental, psychological health
Basic sciences	Improves our understanding of the disease process and aids product development (vaccines, diagnostics and drugs)	•Pathophysiology of disease •Immunology •Product development
Health system	Assists in identifying measures that improve access to the health system and ensures the safety of health workers, and also includes assessment of the impact of COVID-19 on health services or programmes and mitigation measures adopted	•Protection of healthcare workers and infection control in health settings •Service provision including vaccine supply and distribution •Primary healthcare •Impact on other diseases and their control
Public health and social measures	Helps evaluate the usefulness of public health interventions in COVID-19 prevention	•Surveillance and contact tracing •Non-pharmaceutical interventions •Vaccination strategy •Risk communication •Evaluation of legislative approaches
Socioeconomic and equity-related	Helps us understand the impact of COVID-19 on social, economic and equity aspects to plan appropriate interventions	•Community-based approaches •Equity and ethical aspects •Economic impact
Pandemic preparedness	Enables us to strengthen systems to better respond to a “future” pandemic	•Human–animal interface and risk reduction •International reporting/sharing of information •Health system resilience

### Survey to generate research ideas

A webinar was held with WCO research and emergency focal points and SEARO programme managers to orient them to the process and their role. They were all invited to complete an anonymous online survey, which provided the background information on the exercise and its purpose, provided links to earlier prioritization exercises, and described the seven themes mentioned in Table 1. The survey, which was conducted between 25 September and 14 October 2020, was voluntary and asked participants to contribute up to five research questions in their area of expertise. Participants were also allowed to forward the survey invitation to anyone in their office who was involved in the national COVID-19 response.

### Consolidation of research ideas

The research ideas generated by this process were independently reviewed by the core team, with each member being identified as a key discussant for a specific thematic area. Multiple online discussions were held between the members wherein each question was considered for the following possible decisions: retain without change, reformulate and retain, merge with similar idea, split into multiple ideas, or discard. All differences of opinion between members were resolved by discussion to achieve consensus. A proactive effort was made to include gender, equity and human rights considerations when reformulating the ideas.

### Prioritization

The scoring system for prioritization was conducted as a two-step process: identifying the parameters for scoring, followed by finalizing a scoring system. CHNRI lists 15 and ENHR 23 possible parameters for scoring [22, 23]. These were reviewed and six parameters finalized as shown in Table 2. Given the objective of the exercise, regional specificity and urgency (time for results to be available for decision-making) were included in addition to four other parameters of relevance in shaping the pandemic response, feasibility given the regional research capacity, effectiveness or likely impact on clinical or public health practice, and social consideration of gender, equity and human rights. Each parameter was provided with a clarifying statement.

**Table 2:** Scoring system for prioritization of research ideas

Parameter	Explanation	Scoring
Specificity	How specific is the issue to the countries of SEAR?	Very little = 1 Little = 2 Somewhat = 3 Highly = 4
Relevance	How relevant is the research in shaping the pandemic response of countries?	Not relevant = 1 Somewhat relevant = 2 Quite relevant = 3 Highly relevant = 4
Feasibility	How doable is the research idea in the Member States of the region given their technical capacity (institutions, human resource, laboratory, etc.)?	Not doable = 1; Somewhat doable = 2 Quite doable = 3 Highly doable = 4
Urgency	How soon can the results of the research be available for decision-making?	More than 3 years = 1 Within 2–3 years = 2 Within 1 year = 3 Within 6 months = 4
Effectiveness	What impact will the research have on clinical, laboratory or public health practice related to COVID-19 or other health services/programmes?	Very low = 1 Low = 2 High = 3 Very high = 4
Gender, equity and human rights considerations	How likely will the research be to reduce inequities, promote gender responsiveness and advance the right to health?	Not likely = 1 Somewhat likely = 2 Quite likely = 3 Highly likely = 4

All scores were given equal weight. The scoring system followed that recommended by the ENHR approach and began with one point for the first choice, with each following choice scoring one point more than the preceding choice to a maximum of four, giving a maximum total score of 24 for each idea (Table 2). All members first scored independently and then had an open discussion on differing viewpoints, and members were free to change their scoring after that. However, no attempt was made at consensus generation. These individual scores were totalled to get the final score for each idea. These were listed in descending order of the total score, and it was decided that the final prioritized research agenda should not have more than 30 items.

## Results

Invitations for the survey were sent to 56 participants among the attendees of the webinar, and a total of 48 responded to the survey. Seventeen (35%) of the respondents were from SEARO and 31 (65%) from WCOs. Their median period of service within WHO was 5.5 years. The participants were mainly from communicable diseases (n=16), WHO health emergencies (n=15) and health systems and life course (n=11) departments. All countries except the Democratic People’s Republic of Korea responded, with maximum response from Myanmar (n=11).

A total of 203 research ideas were received through the survey. Seventy-three (73, 36%) of these ideas came from SEARO respondents and the rest (130, 64%) from WCOs. Most ideas were related to the health system (n=56), epidemiology (n=44) and public health (n=43) themes, predominantly driven by responses from SEARO and Myanmar (Table 3).

**Table 3:** List of research ideas by thematic areas and sources of suggestion

WHO country/regional office	Basis sciences	Epidemiology	Clinical sciences	Health system	Public health interventions	Pandemic preparedness	Social, economic and equity-related	Total
Bangladesh	1	5	3	3	5	2	1	20
Bhutan	0	1	1	2	0	0	0	4
India	1	4	2	–	1	0	2	10
Indonesia	0	2	–	3	3	0	–	8
Maldives	1	1	–	2	1	0	0	5
Myanmar	1	5	6	14	8	1	3	38
Nepal	0	9	2	2	5	2	0	20
Sri Lanka	1	4	1	–	0	1	0	7
Thailand	0	3	0	1	4	0	1	9
Timor-Leste	0	1	0	5	2	0	1	9
Regional office	2	9	11	24	14	5	8	73
Preliminary list (%)	7 (3.4)	44 (21.7)	26 (12.8)	56 (27.6)	43 (21.2)	11 (5.4)	16 (7.9)	203
Shortlist (%)	4 (5.1)	17 (21.8)	12 (15.4)	23 (29.5)	13 (16.7)	5 (6.4)	4 (5.1)	78
Priority list (%)	1 (3.7)	5 (18.5)	1 (3.7)	10 (37.0)	6 (22.2)	1 (3.7)	3 (11.1)	27

During the process of consolidation, it was noted that the interaction of COVID-19 with other diseases formed the bulk of the ideas in three of the thematic areas. Under the clinical theme, most questions were related to the bidirectional impact of COVID-19 and other diseases (impact of COVID-19 on other diseases like diabetes and tuberculosis outcomes, and of these diseases on COVID-19 outcomes). Under the epidemiological theme, the bidirectional impact of COVID-19 and other diseases on epidemiology, transmission and burden including mortality of either was most frequent. Under the health systems theme, most ideas were related to the surge capacity of different health system dimensions to respond effectively to COVID-19 or health system capacity to keep key COVID-19 and non-COVID-19-related health services going—extent of disruption of various types of health services and measures taken to ensure the continuity of these services.

After discussion among the core group members, the research ideas were retained with or without rephrasing (*n* = 43), merged with another related or similar idea (*n* = 156), or dropped as not considered a research question (*n* = 4). This process resulted in a final list of 78 research ideas. In this list, health system (n=23) and epidemiology (n=17) continued to have the highest number of research ideas.

After arranging the sum of the scores given by the core group members in descending order, it was decided to consider the upper tertile as of “higher priority”. This was to keep the maximum number of priority research ideas below 30. The final 27-item priority research agenda covered all the thematic areas—health system (n=10), public health and social measures (n=6), epidemiology (n=5), socioeconomic and equity (n=3), basic sciences (n=1), clinical sciences (n=1) and pandemic preparedness (n=1) (Table 4).

**Table 4:** Priority research agenda for COVID-19 in SEAR

Rank	Research idea	Contributing WHO offices
*Thematic area: health system*		
1	What is the effect of the COVID-19 pandemic on the continuity of essential health services?	Bhutan, Nepal, SEARO
2	What are the major gaps in policies and strategies in effectively responding to the pandemic in SEAR Member States?	SEARO
3	How was the readiness for the delivery of COVID-19 vaccines assessed in SEAR Member States; what were the key aspects where the countries were ready and where were the gaps?	Myanmar
5	What was the impact of COVID-19 on healthcare workers (including rates of infection, stress, burnout, stigmatization and violence) and measures taken to address them, including any gender-specific response?	Bangladesh, Indonesia, Myanmar, Nepal, Timor-Leste, SEARO
7	Assessment of health system capacity (especially preventive and promotive) to manage the epidemic	Myanmar, Timor-Leste, SEARO
11	What was the role of the primary level of care in SEAR Member States in case management and its referral linkages with higher levels?	Myanmar, Timor-Leste, SEARO
13	In SEAR Member States, how did the existing roles and responsibilities of the hierarchy in the governance system of COVID-19 response enable or constrain an effective pandemic response?	Myanmar
14	How were supply chain issues quantified and reliable supplies ensured from national to subnational levels in SEAR Member States for all essential items?	SEARO
17	To what extent were health services (diagnostic, curative, promotive, preventive, rehabilitative) disrupted due to COVID-19 and what was the resulting adverse impact on the prevention and control of priority public health conditions (TB, mental health, SRMNCAH, NCDs)?	Bhutan, Myanmar, Thailand, SEARO
24	What process changes did SEAR Member States introduce in healthcare facilities to cater to the dual load of COVID-19 and non-COVID-19 patients during the pandemic?	Maldives
*Thematic area: public health and social measures*		
6	Acceptability of COVID-19 vaccine and barriers to vaccination among the general population and among healthcare workers	Timor-Leste, SEARO
8	What are the sociocultural influences and other barriers to and enablers of community behaviour change regarding COVID-19?	Bangladesh, Indonesia, Myanmar, Timor-Leste, SEARO
12	What policy and programmatic interventions for the COVID response are safe and effective in preventing transmission in different local contexts (points of entry/internally displaced people/workplaces)?	Bangladesh, India, Maldives, Myanmar, Nepal, Thailand, SEARO
21	Analysis of prevention strategies by SEAR Member States and their effectiveness in controlling the transmission of COVID-19 and limiting its adverse socioeconomic impact	Bangladesh, India
25	Analysis of the use of contact tracing mobile applications to accelerate the COVID-19 response and for addressing challenges in case finding and contact tracing in communities, in different contexts within countries	Bangladesh, Indonesia, Myanmar, Nepal, SEARO
26	Evaluation of the COVID-related communication campaigns in SEAR Member States	Bangladesh, Myanmar, SEARO
*Thematic area: epidemiology*		
4	To estimate the severity of disease (mild/moderate/severe) in various groups (by age group, geographical location, sex, health status, vulnerable groups, etc.) and conduct a trend analysis	Bangladesh, Myanmar, Nepal, Thailand, Timor-Leste, SEARO
10	Sero-surveillance/sero-surveys to assess and monitor the infection burden of SARS-CoV-2 infection in various groups (by age, geographical location, sex, etc.)	Myanmar, Nepal
16	What are the early warning indicators to identify COVID-19 clustering?	Sri Lanka
23	COVID-19 disease transmission studies in various settings (slums, rural areas, workplaces)	Bangladesh, India, Sri Lanka
27	Analysis of COVID-19 data disaggregated by sex and age (and any other available stratifier) for policy-relevant trends revealed by such analysis	Bangladesh, Myanmar
*Thematic area: socioeconomic and equity*		
9	How did COVID-19 exacerbate pre-existing inequities and their (negative) health and non-health impact on various disadvantaged groups, taking as migrants or others as illustrative examples?	Indonesia, Myanmar, Timor-Leste, SEARO
19	To what extent have sex and gender figured/been addressed in clinical trials and other COVID-19-related research in SEAR Member States?	Nepal, SEARO
22	Which COVID-19 related policies have addressed the needs of vulnerable groups and to what extent has the response been equity-focused, gender-responsive and human rights-based (using specific vulnerable groups as illustrative examples)?	Bangladesh, India, Myanmar, Thailand, Timor-Leste
*Thematic area: clinical sciences*		
15	Clinical features, disease progression and outcome of COVID-19 infection in various vulnerable groups (by age, sex or health status, migrants, refugees, internally displaced persons, slum-dwellers, people living with a disability, etc.)	Bangladesh, Bhutan Indonesia, Myanmar, Nepal, Sri Lanka, Thailand, SEARO
*Thematic area: basic sciences*		
18	What is the duration and level of immune response in COVID-19 positive patients by age, sex and comorbidities (HIV/TB/NCDs)?	Bangladesh, Maldives, Sri Lanka, Timor-Leste, SEARO
*Thematic area: pandemic response*		
20	How did existing laboratory capacity influence the national testing strategy adopted in SEAR Member States; how did countries improve their laboratory capacity, and what lessons have been learned to improve laboratory performance in the future?	Bangladesh, Nepal

*NCDs* noncommunicable diseases, *SRMNCAH* sexual, reproductive, maternal, newborn, child and adolescent health, *TB* tuberculosis

The key research priorities identified on the health system theme were “measuring the effect of COVID-19 on essential health services”, “ability to manage dual (COVID-19 and non-COVID-19) load of patients”, “supply chain and vaccine delivery aspects”, “healthcare worker safety”, and “health system capacity” and “governance”. The public health and safety measures (PHSM)-related research priorities included “evaluation of effectiveness of different interventions for prevention of transmission”, “contact tracing strategies and their effectiveness” and barriers to roll out of preventive strategies such as vaccination and behavioural interventions. Under the epidemiology theme, ideas related to burden including serology, disease transmission, thresholds for early warning and disaggregated analysis of data were identified. The worsening of inequities due to COVID-19 and the equity focus of national COVID-19 responses were identified as key research areas in the socioeconomic and equity thematic area.

## Discussion

Each research priority-setting exercise is unique and is tailored to its context in terms of method of generation and prioritization of research ideas. This exercise was conducted with a clear focus on the public health aspects that shape national COVID-19 responses. It has resulted in identifying key research ideas relevant to the region. This exercise followed standard guidance and used open and transparent procedures, including independent scoring rather than consensus. Key limitations were largely driven by the short time frame of 3 months from conception to execution, which precluded a fuller consultative process of involvement of national-level stakeholders. However, given the urgency of the exercise, this is not unexpected. The respondents for the survey were WHO staff, selected based on their involvement with the national pandemic response and likely familiarity with country evidence priorities and gaps. The prioritization was done by a small group, with the possibility of bias by their own research interests, which was partly compensated by independent scoring and the diverse backgrounds of the core group. It is acknowledged that this list was not intended to cover all the research that needs to be undertaken, such as environmental issues, mental health or digital health interventions and many more.

There are various ways to identify research priorities, each with its advantages and disadvantages. The key issues in any such exercise are the process of generating research ideas, criteria for scoring and the prioritization process. Research idea generation could be by a review of literature or a survey of experts and sometimes through large stakeholder consultation. The timeline for such processes also varies from several months to a few weeks. The size, composition and process for selecting experts determines the validity of this step. In previous COVID-19 research prioritization exercises, the size of the group has varied from above 1500 to 5, as compared with 48 in this exercise [8, 13]. All have adopted online surveys, reflecting the COVID-19 realities. Scoring methods to identify priorities have varied from experts conducting a simple scoring on a scale of 1–10 for each topic or experts each choosing their top three research ideas, to a detailed scoring on pre-identified parameters, as was done in this exercise [8]. Finally, the composition of the group of people who do the scoring for prioritization impacts the prioritization process and reflects their bias. Most prioritization exercises have involved researchers and academicians as experts rather than users of the research as in this case.

This process identified health system and public health issues as major research priorities in the current period, as these are downstream issues inherently linked to national responses. While earlier such exercises had an epidemiological and clinical focus, in this exercise the role of health system and equity in national COVID-19 responses gained importance [24–26]. This may reflect evolving priorities over time in the current pandemic as well as the agency conducting the prioritization exercise. In a priority listing of research in the health policy and systems research (HPSR) space, Gilson et al. identified the following areas: differential impact of COVID-19 on disadvantaged groups, impact on local health system by COVID-19, and governance and decision-making [26]. Other researchers have made recommendations related to the need to protect essential services during the pandemic [27]. Similarly, recommendations have been made for an equity-driven response, applying a social determinants and health equity lens on monitoring, evaluation and clinical trials; and the need to dedicate resources to prioritize high-risk communities for clinical management and prevention as well as policies that address the social and economic barriers that these populations face during a pandemic [28]. Our exercise reinforces these recommendations.

While prioritization is an important step, the more critical step is the implementation of the prioritized research agenda. WHO has a role in convening stakeholders to set research priorities, but is usually not engaged in implementation of research. This exercise would help in building a COVID-19 research roadmap for WHO SEARO and its Member States which will ensure efficient use of limited resources available for COVID-19 research. WHO (headquarters/SEARO/country office) technical staff and senior management can use the priorities thus generated in policy dialogue and advocacy with Member States and in the development of their work plans and resource allocation decisions. The prioritized list may also act as a catalyst for Member States and other stakeholders including development partners to support research which could help shape the COVID-19 response in SEAR. When taking up these ideas for implementation, researchers may need to formulate their questions more specifically with reference to their own country or programme area. It is also recommended that they reflect gender, equity and human rights concerns in formulating their specific questions. This priority agenda should also stimulate an assessment of the research capacity of Member States to address these concerns and measures to strengthen the response, including greater investment in health research (from basic sciences to translational) and the promotion of national and international research collaborations.

In conclusion, this exercise has been a timely effort to identify current public health research priorities that are needed for countries to respond more effectively to the COVID-19 pandemic. It should also be noted that the dynamic nature of the pandemic means that the priorities will change over time, and this process may need to be repeated in due course.

## Data Availability

The datasets used and/or analysed during the current study are available from the corresponding author on reasonable request.
